# Lipid Levels and 3-Month Prognosis After Spontaneous Intracerebral Hemorrhage in Women

**DOI:** 10.3389/fneur.2021.690194

**Published:** 2021-06-17

**Authors:** Hao Feng, Xin Wang, Wenjuan Wang, Xingquan Zhao

**Affiliations:** ^1^Department of Neurology, Beijing Tiantan Hospital, Capital Medical University, Beijing, China; ^2^China National Clinical Research Center for Neurological Diseases, Beijing, China; ^3^Research Unit of Artificial Intelligence in Cerebrovascular Disease, Chinese Academy of Medical Sciences, Beijing, China

**Keywords:** spontaneous intracerebral hemorrhage, 3-month prognosis, women, total cholesterol, non-high-density cholesterol, low-density cholesterol, triglycerides, high-density cholesterol

## Abstract

**Background:** The relationship between serum lipids levels and prognosis after spontaneous intracerebral hemorrhage (ICH) is still unclear. We aim to examine the association between lipid levels and 3-month ICH prognosis in women.

**Method:** We went through a registry of spontaneous ICH cases and selected female patients to study according to our criteria. We collected demographic, clinical, and laboratory information and evaluated serum triglyceride (TG) levels, total cholesterol (TC) levels, low-density cholesterol (LDLC) levels, high-density cholesterol (HDLC) levels, non-high-density cholesterol (non-HDLC) levels, and 3-month modified Rankin Scale (mRS). Multivariate logistic regression was performed, and receiver operating characteristic (ROC) curves were plotted to explore the relationship between serum lipid levels and 3-month ICH clinical outcomes.

**Results:** Two hundred six female patients were included in this study, and 96 (46.6%) of them had poor functional outcomes. In the univariate analysis, low TG (*p* = 0.006), TC (*p* = 0.025), LDLC (*p* = 0.001), non-HDLC (*p* < 0.001) levels, and high HDL (*p* = 0.036) levels were associated with poor 3-month clinical outcomes in women. In the multivariate logistic regression, low levels of TG (OR = 0.711, 95% CI = 0.542–0.933, *p* = 0.014), TC (OR = 0.523, 95% CI = 0.304–0.903, *p* = 0.020), LDLC (OR = 0.538, 95% CI = 0.307–0.942, *p* = 0.030), non-HDLC (OR = 0.327, 95% CI = 0.177–0.603, *p* < 0.001), and a high level of HDLC (OR = 2.075, 95% CI = 1.064–4.047, *p* = 0.032) with area under the curve (AUC) of 0.610, 0.590, 0.630, 0.645, and 0.415, respectively, remained as independent indicators of poor prognosis at 3 months after adjusting for confounding factors.

**Conclusion:** Low levels of TG, TC, LDLC, non-HDLC, and high levels of HDLC were independently associated with poor prognosis of spontaneous ICH in women.

## Introduction

Spontaneous intracerebral hemorrhage (ICH) is a neurological disease with a high mortality and morbidity (1). The incidence rate was about 10 to 30 per 100,000 people (2). According to the global, regional, and country-specific lifetime risk of stroke 1990–2016, Chinese men have the greatest lifetime stroke risk [41.1% (39.2–42.9)], and the difference in risk between men [41.1% (39.2–42.9)] and women [36.7% (35.0–38.6]) was also the largest (3). Stroke is the third leading cause of death in China (4). All over the world, ICH accounts for 10–15% of total strokes each year. The fatality rate within 1 month after ICH can be as high as 40.4% (1). Only a small proportion (12–39%) of survivors could achieve functional independence (5).

While high levels of serum total cholesterol (TC) and low-density cholesterol (LDLC) are closely related to an increased risk of ischemic stroke (6–8), the association between lipoprotein cholesterol levels and intracerebral hemorrhage (ICH) remains controversial. In the Korea Medical Insurance Corporation Study, a low TC level was not an independent risk factor for ICH in 114,793 men (9). However, in a prospective study of the relationship between serum total cholesterol and stroke incidence, it was found that higher serum TC levels in women often indicated a lower risk of ICH. After adjusting for confounding factors, the hazard ratios (HR) for different levels of TC [<5(reference), 5–5.9, 6–6.0, and ≥7.0 mmol/L) were 1.00, 0.58, 0.61, and 0.50, respectively (p trend = 0.02) (10). Since lipid-lowering strategies are widely used for the prevention of cardiovascular diseases, we are interested in whether low lipid levels will increase ICH risk. Previous studies observed that low serum lipid levels may increase the mortality of ICH patients (11–14); however, a few studies have focused on the relationship between women's serum lipid levels and the occurrence and prognosis of ICH. Studies have shown that women have a higher burden of stroke compared with men (15), which means that it is particularly important to be concerned of the risk factors among women. Currently, there has not been any research on the association between serum lipid level and the prognosis of female patients.

Therefore, in this study, we aim to identify the association between serum lipid levels, including TC, LDLC, triglycerides (TG), non-high-density cholesterol (non-HDLC), and HDLC, and the prognosis of ICH in a prospective cohort of women in this study.

## Methods

All patients participating in this study provided written informed consent. The study protocol was approved by the Institutional Review Board of the Beijing Tiantan Hospital affiliated to Capital Medical University, and this study was approved by the Beijing Tiantan Hospital Ethics Committee.

Our analysis was performed using data from a multicenter, prospective, and observational cohort study (Registration Study on the Medical Quality Evaluation of Cerebral Hemorrhage Based on Etiology in the Beijing Area. The main aim of that cohort study was to analyze the gender and age distribution of different ICH etiologies and the medical conditions of ICH in the Beijing area). A total of 13 hospitals in Beijing participated in the study. Eventually, 1,964 eligible ICH patients were included in the database (16). The design of the database was outlined as described by Feng et al. (16). For our study, we specifically focused on female patients in the ICH database.

### Inclusion and Exclusion Criteria

**Figure 1:**
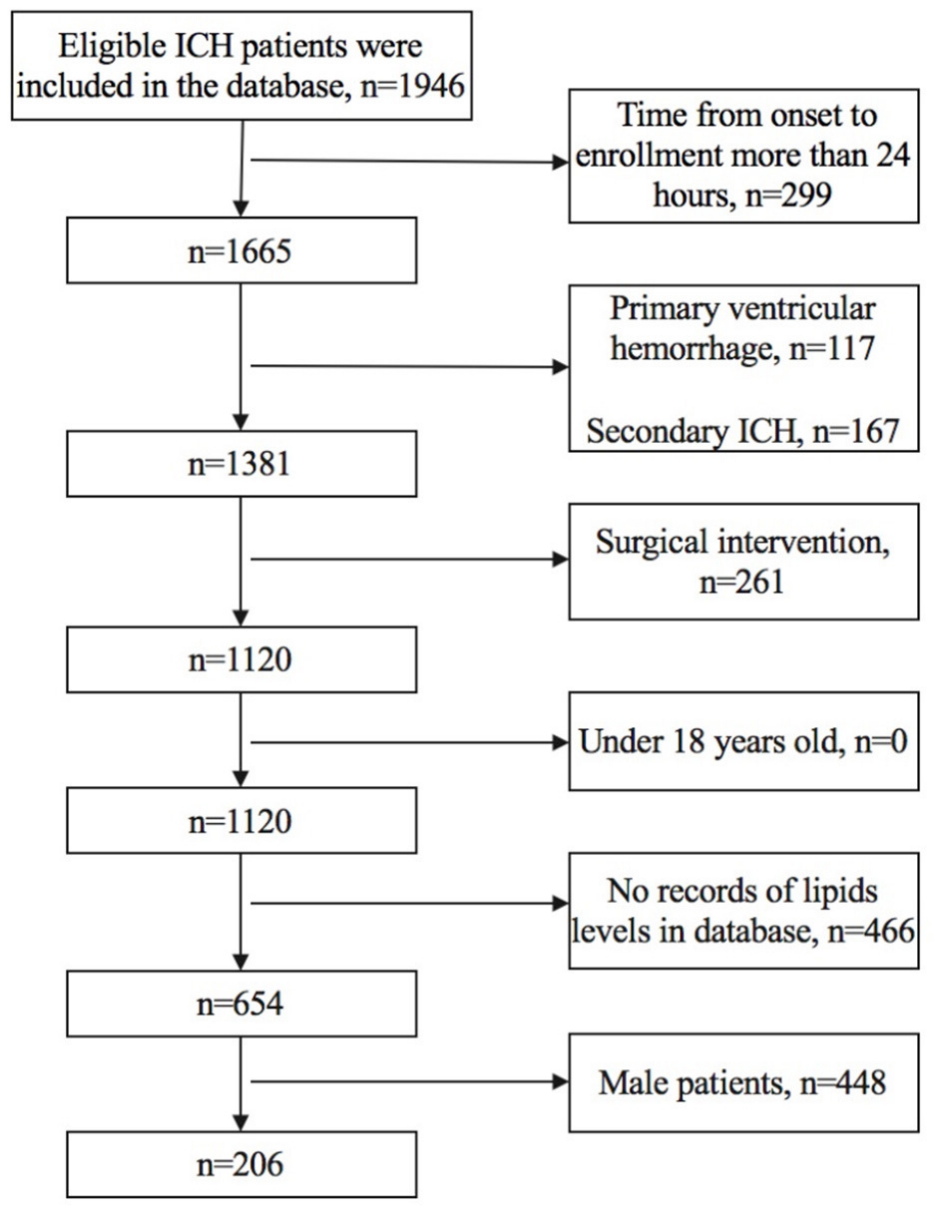
Flowchart of patients' selection.

We included female patients whose (1) time from onset to enrollment was not more than 24 h, (2) TG, TC, LDLC, HDLC, and non-HDLC levels were all documented in the database, and (3) age was older than 18 years old. We excluded patients who had (1) primary ventricular hemorrhage; (2) diagnosis of secondary ICH, including head trauma, brain tumor, aneurysm, cavernous hemangioma, arteriovenous malformations, acute thrombolysis, coagulopathy, and moyamoya disease; (3) surgical intervention, including extraventricular drainage, craniotomy, hematoma puncture, and aspiration during the follow-up; and (4) anticoagulant therapy before the onset of symptoms. Figure 1 shows details regarding the selection of patients.

### Baseline Information

By using standard questionnaires, trained stroke physicians collected demographic information, past medical history, and past medications for each enrolled patient, and conducted clinical evaluations of the patients' neurological function. Arterial hypertension was diagnosed when documented in medical records or when at least two blood pressure readings were higher than 140 mmHg (systolic) or 90 mmHg (diastolic) after the acute phase of stroke. Diabetes was recorded when a patient had known diabetes mellitus at presentation, or the plasma glucose level was higher than 11 mmol/L on admission or during hospital stay. Cigarette smoking was classified as never, previous, or current smokers based on daily tobacco consumption. Alcohol intake was classified as current drinker or non-drinker.

The variables that were collected included age, past medical history, National Institutes of Health Stroke Scale (NIHSS) at admission, Glasgow Coma Scale (GCS) at admission, blood pressure level at admission, and secondary intraventricular hemorrhage. On the initial CT scan, we evaluated ICH localization and volume (ABC/2 method). Routine blood sampling and testing were performed within 1 h of the patients' arrival. Fasting blood samples were collected from an antecubital vein on the morning after arrival following an overnight fasting (>8 h), and serum TG, TC, HDLC, and LDLC levels were recorded. Non-HDLC levels were calculated by subtracting serum HDLC levels from serum TC levels. We also documented prior antiplatelet/statin use and statin use after admission.

### Follow-Up Information and Intracerebral Hemorrhage Clinical Outcomes

The 3-month modified Rankin Scale (mRS) scores of the patients were assessed *via* telephone interviews. The interviewers were trained on standard protocol and were blinded to the patients' clinical, laboratory, and imaging information during the follow-up evaluation. If we were not able to reach the patients on the first telephone interview attempt, we then telephoned them once a week for up to three more weeks. The patient was considered unreachable if none of the four telephone interview attempts were successful. An mRS ≥3 was regarded as a poor prognosis, and mRS <3 was regarded as a good prognosis.

### Statistical Analyses

For continuous variables, we use mean values together with their corresponding standard deviations (SDs) to describe the normally distributed data, and median [interquartile range (IQR)] to describe non-normally distributed data. Continuous variables were compared using Student's *t*-test or Mann–Whitney *U*-test as appropriate. Chi-squared test was applied to compare categorical variables. Multivariate analysis with the enter method logistic regression model was performed to determine the independent predictors. Receiver operating characteristic curves (ROC) were plotted to assess the discriminative predictive value of the lipid levels. The areas under the receiver operating characteristic curves (AUC) were calculated and compared. All tests of significance were two tailed. A *p*-value < 0.05 was set as the significance level for all analyses. SPSS software (version 24.0; IBM, Armonk, NY, USA) was the software program used to perform the statistical analyses.

## Results

We finally included 206 female patients with spontaneous ICH (Figure 1). The median age was 62 (52–71) years old, ranging from 25 to 91 years old, 96 patients (46.6%) had poor prognosis at 3 months (mRS score of 3–6), the median time from onset to admission was 2.87 (1.50–5.67) h, 18 patients (8.7%) had hyperlipidemia, and 10 (4.9%) patients had prior statin use before admission. The median volume of hematoma was 8.85 (4.10–18.68) ml, and 178 (86.4%) subjects had supratentorial ICH (Table 1).

**Table 1:** Comparison of characteristics between female patients with good and poor 3-month clinical outcome.

	**Total**	**Good outcome (mRS <3)**	**Poor outcome (mRS ≥ 3)**	***p*-Value**
Number (%)	206	110 (53.4)	96 (46.6)	/
Age (years, median, IQR)	62 (52–71)	56 (50–65)	67 (55–76)	<0.001
Onset to admission time (h, median, IQR)	2.87 (1.50–5.67)	3.00 (1.77–6.05)	2.62 (1.43–4.83)	0.133
Hypertension no. (%)	160 (77.7)	87 (79.1)	73 (76.0)	0.600
Diabetes mellitus no. (%)	36 (17.5)	18 (16.4)	18 (18.8)	0.653
Hyperlipidemia no. (%)	18 (8.7)	10 (9.1)	8(8.3)	0.848
History of CI no. (%)	31 (15.0)	14 (12.7)	17 (17.7)	0.319
History of ICH no. (%)	7 (3.4)	3 (2.7)	4 (4.2)	0.855
Smoking no. (%)	8 (3.9)	3 (2.7)	5 (5.2)	0.358
Alcohol no. (%)	14 (6.8)	7 (6.4)	7 (7.3)	0.792
Prior antiplatelet use no. (%)	35 (17.0)	14 (12.7)	21 (21.9)	0.081
Prior statin use no. (%)	10 (4.9)	4 (3.6)	6 (6.3)	0.384
NIHSS score on admission (median, IQR)	9 (3–15)	5 (1–9)	13 (8–20)	<0.001
GCS score on admission (median, IQR)	14 (12–15)	15 (14–15)	13 (10–15)	<0.001
SBP on admission (mmHg, median, IQR)	164 (150–184)	165 (150–180)	163 (147–188)	0.913
DBP on admission (mmHg, median, IQR)	95 (81–104)	96 (83–104)	90 (80–104)	0.322
Glucose on admission (median, IQR)	7.18 (6.20–9.38)	6.98 (6.13–9.03)	7.21 (6.40–9.59)	0.259
Fasting blood glucose (median, IQR)	5.88 (5.02–8.01)	5.51 (4.62–7.12)	6.46 (5.45–8.48)	0.001
WBC on admission [1,000 (median, IQR)]	8.04 (6.32–11.12)	7.83 (6.19–10.08)	9.05 (6.52–12.30)	0.010
Platelets on admission [1,000 (mean ± SD)]	218 ± 56	220 ± 53	215 ± 59	0.439
INR (median, IQR)	0.96 (0.91–1.01)	0.95 (0.91–1.00)	0.97 (0.93–1.04)	0.037
Creatinine on admission (mmol/L median, IQR)	52.0 (44.6–62.0)	51.1 (43.4–59.0)	53.0 (46.1–65.0)	0.068
In hospital TG (mg/dl, median, IQR)	105.00 (75.31–150.18)	116.07 (78.85–162.80)	92.59 (67.56–133.34)	0.006
In hospital TC (mg/dl, median, IQR)	182.28 (160.22–209.17)	190.60 (164.67–217.69)	177.25 (155.96–201.34)	0.025
In hospital LDLC (mg/dl, median, IQR)	112.04 (91.33–135.55)	117.84 (98.30–142.61)	103.91 (87.46–124.23)	0.001
In hospital HDLC (mg/dl, median, IQR)	50.50 (41.02–62.69)	48.57 (41.02–57.37)	53.79 (41.89–66.76)	0.036
In hospital non-HDLC (mg/dl, median, IQR)	128.29 (108.26–156.44)	138.93 (113.97–169.12)	122.87 (98.01–142.71)	<0.001
Statin use after admission no. (%)	23 (11.2)	15 (13.6)	8 (8.3)	0.228
Hematoma volume (ml, median, IQR)	8.85 (4.10–18.68)	6.39 (3.00–12.13)	12.10 (6.68–24.40)	<0.001
Location				0.040
Supratentorial no. (%)	178 (86.4)	90 (81.8)	88 (91.7)	/
Infratentorial no. (%)	28 (13.6)	20 (18.2)	8 (8.3)	/
sIVH no. (%)	64 (31.1)	25 (22.7)	39 (40.6)	0.006

*CI, cerebral infarction; DBP, diastolic blood pressure; GCS, Glasgow Coma Scale; HDLC, high-density lipoprotein cholesterol; ICH, intracerebral hemorrhage; IQR, interquartile range; LDLC, low-density lipoprotein cholesterol; mRS, modified Rankin Scale; NIHSS, National Institutes of Health Stroke Scale; non-HDLC, non-high-density lipoprotein cholesterol; SBP, systolic blood pressure; sIVH, secondary intraventricular hemorrhage; TC, triglycerides; TG, total cholesterol; WBC, white blood cells*.

### Predictors of Poor 3-Month Outcome in Female Patients With Spontaneous Intracerebral Hemorrhage Patients

Female ICH patients who had poor 3-month prognosis (mRS ≥ 3) were older (*p* < 0.001) and had higher NIHSS (*p* < 0.001), lower GCS (*p* < 0.001), larger hematoma volume (*p* < 0.001), higher proportion of secondary intraventricular hemorrhage (*p* < 0.001), higher levels of fasting blood glucose (*p* = 0.001), higher levels of white blood cell count (WBC) on admission (*p* = 0.010), and higher international normalized ratio (INR) (*p* = 0.037). We found that female ICH patients with mRS ≥ 3 had increased levels of HDLC (*p* = 0.036) and decreased levels of TC (*p* = 0.025), TG (*p* = 0.006), LDLC (*p* = 0.001), and non-HDLC (*p* < 0.001). There was no significant association between statin use and 3-month prognosis (Table 1).

In multivariate logistic analysis (Table 2), low levels of TG (OR = 0.711, 95% CI = 0.542–0.933, *p* = 0.014), TC (OR = 0.523, 95% CI = 0.304–0.903, *p* = 0.020), LDLC (OR = 0.538, 95% CI = 0.307–0.942, *p* = 0.030), non-HDLC levels (OR = 0.327, 95% CI = 0.177–0.603, *p* < 0.001), and high levels of HDLC (OR = 2.075, 95% CI = 1.064–4.047, *p* < 0.032) remained as independent indicators of poor clinical outcomes after adjusting for age, NIHSS, GCS scores on admission, fasting blood glucose, WBC on admission, INR on admission, hematoma volume, hematoma location, and secondary intraventricular hemorrhage.

**Table 2:** Multivariate-adjusted^#^ OR and 95% CI of lipid levels for poor 3-month outcome (mRS ≥ 3).

	**OR (95% CI)**	***p*-Value**
TG (50 mg/dl)	0.711 (0.542–0.933)	0.014^*^
TC (50 mg/dl)	0.523 (0.304–0.903)	0.020^*^
HDLC (25 mg/dl)	2.075 (1.064–4.047)	0.032^*^
LDLC (50 mg/dl)	0.538 (0.307–0.942)	0.030^*^
Non-HDLC (50 mg/dl)	0.327 (0.177–0.603)	<0.001^*^

*CI, confident interval; HDLC, high-density lipoprotein cholesterol; LDLC, low-density lipoprotein cholesterol; mRS, modified Rankin Scale; non-HDLC, non-high-density lipoprotein cholesterol; TC, triglycerides; TG, total cholesterol; OR, odd ratio.*

# *Adjusted for age, NIHSS, and GCS scores on admission, fasting blood glucose, WBC on admission, INR on admission, hematoma volume, hematoma location, and secondary intraventricular hemorrhage.*

* *p < 0.05*.

### Sensitivity and Specificity Tests of Lipid Levels for 3-Month Clinical Outcome in Female Patients With Spontaneous Intracerebral Hemorrhage Patients

**Figure 2:**
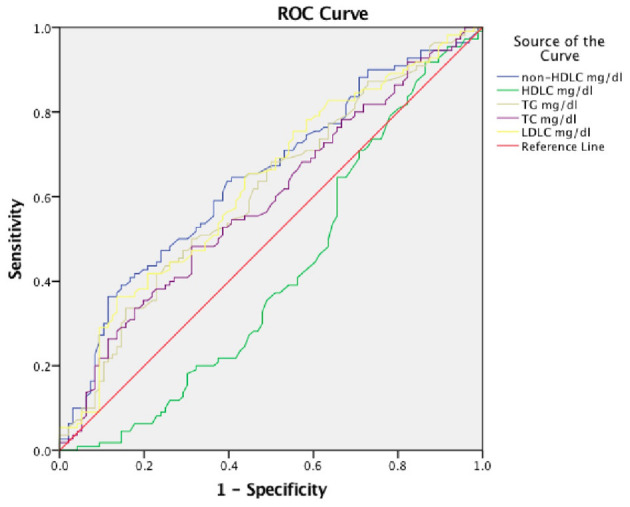
Receiver operating characteristic (ROC) curves for 3-month prognosis according to lipid profiles. TC, total cholesterol; LDLC, low-density lipoprotein cholesterol; non-HDLC, non-high-density lipoprotein cholesterol; TG, total cholesterol; HDLC, high-density lipoprotein cholesterol.

We further, studied the accuracy of the predictive value and the cutoff value of the clinical outcomes (Table 3). The sensitivity and specificity of TG, TC, LDLC, non-HDLC, and HDLC were 41.8, 47.3, 36.4, 36.4, and 91.8%, and 22.9, 68.7, 86.5, 88.5, and 13.5%, respectively. The ROC was generated (Figure 2), and the AUCs were calculated for TG, TC, LDLC, non-HDLC, and HDLC in relation to clinical outcomes (0.610, 0.590, 0.630, 0.645, and 0.415, respectively; Table 3). Although, the AUC of TG reached 0.61, its sensitivity and specificity were very low (41.8 and 22.9%). The sensitivity of HDLC was as high as 91.8%, but its specificity was very low (13.5%). Compared with LDLC levels and TC levels, non-HDLC levels have higher predictive value for 3-month clinical outcomes in female patients with spontaneous ICH.

**Table 3:** Predictive abilities of lipid levels for clinical outcomes.

	**AUC**	***p***	**Cutoff**	**Sensitivity (%)**	**Specificity (%)**
TG	0.610	0.006	135.56	41.8	22.9
TC	0.590	0.025	193.5	47.3	68.7
LDLC	0.630	0.001	135.06	36.4	86.5
non-HDLC	0.645	<0.001	156.54	36.4	88.5
HDLC	0.415	0.036	36.96	91.8	13.5

*AUC, area under the curve; TC, total cholesterol; LDLC, low-density lipoprotein cholesterol; non-HDLC, non-high-density lipoprotein cholesterol; TG, total cholesterol; HDLC, high-density lipoprotein cholesterol*.

## Discussion

Our study evaluated the association between serum lipid levels in female ICH patients and the 3-month prognosis. Our results suggested that low TG, TC, LDLC, non-HDLC levels, and high HDLC levels in female patients indicated a poor prognosis at 3 months.

Although, previous literatures have reported the relationship between serum lipid levels and the risk of ICH (13, 14, 17), a few studies have explored whether serum lipid levels affect the prognosis of patients with ICH. In a previous study of 2,444 ICH patients from Taiwan, patients with low TC levels (<160 mg/dl) were more likely to have a poor 90-day prognosis (OR = 1.41, 95% CI = 1.11–1.78) (18). Another study found that ICH patients with low serum TC levels (≤ 150 mg/dl) had their mRS scores worsen (OR = 3.3, 95% CI = 1.33–8.00) at 30 days from onset (19). Excessive lowering of serum TC levels may cause unfavorable clinical outcomes in ICH recovery (19). In our study, the cutoff TC predictive value for ICH poor prognosis was 156.54 mg/dl, and this finding suggested that patients with TC levels lower than 156.54 mg/dl may have a poor prognosis. Our results were consistent with previous studies, which found that low serum lipid levels were associated with poor ICH outcome.

In 2009, Ramirez-Moreno et al. (11) suggested that low LDLC levels were independently associated with death after ICH (HR = 3.07, 95% CI = 1.04–9.02, *p* = 0.042). The association between low LDLC levels and the death after ICH was confirmed by other studies (12, 20, 21). A previous study suggested that low LDLC levels were not associated with poor mRS after ICH (12); however, we found that low-level LDLC was an independent risk factor for poor prognosis in female patients after ICH. The difference could be caused by differences in population and gender. In our study, we also reported the relationship between low non-HDLC levels and poor prognosis in female patients with ICH, which was consistent with our previous study where we analyzed ICH patients of both genders (16). In our study, we found that low TG levels and high HDLC levels were associated with poor prognosis in female patients, and this result conflicted with previous studies (12, 18, 22, 23). The reason might be that TG and HDLC only affected the prognosis of female ICH patients and had no association with the prognosis of male ICH patients, while previous studies did not specifically analyze female patients.

A study by Zhang et al. (10) showed that TC levels were positively correlated with the risk of all types of stroke and ischemic stroke in men, and were negatively correlated with the risk of ICH in women. They did not find any association between TC levels and ICH risk in men. This reminded us that the effect of cholesterol levels on blood vessels in men and women may vary, and the mechanism deserves further study. The most common cause of ICH was microaneurysm ruptures caused by hypertension, and the most vulnerable arteries were the penetrating arteries because those arterial walls sustain higher stresses. Previous studies had put forward the inference that low levels of serum cholesterol may reduce atherosclerosis in large proximal intracranial arteries, which exposed the distal penetrating arteries to higher wall stress (24). Based on the above theory, we can speculate that the distal penetrating artery around the hematoma in ICH patients with low baseline cholesterol levels is more prone to ischemic damage, which leads to a poorer clinical prognosis.

We demonstrated that low non-HDLC levels and low LDLC levels predicted poor clinical outcomes in female ICH patients with high specificity. Although, HDLC had a high sensitivity, its specificity was so low that we surmised that it had no clinical predictive value. The sensitivity and specificity of TG were very low; thus, we considered that it did not have high clinical value in predicting prognosis.

In our study, we did not find significant differences in the statin use between the poor and good prognosis groups. Statins were mainly used for the prevention of ischemic cerebrovascular diseases. Lipid-lowering therapy was associated with an increased ICH risk in secondary prevention trials (OR = 1.18, 95% CI = 1.00–1.38), but not in primary prevention trials (OR = 1.01; 95% CI = 0.78–1.30). The benefits of lipid-lowering therapy in preventing ischemic stroke greatly outweighed the possible increased risk of ICH. Based on our study results, stroke clinicians do not need to refrain from prescribing lipid-lowering treatments for secondary prevention of ischemic stroke due to concerns about increased risk of cerebral hemorrhage is not recommended (25).

There were some potential limitations in our study. First, we did not continuously monitor changes in serum lipid levels during follow-up continuously. The patients' cholesterol levels may have varied over time and that may have impacted the prognosis. Second, the percentage of pre-ICH lipid-lowering therapy was too small. Finally, we recruited patients from the emergency department, and we were unable to complete the collection of the blood lipid information for some patients due to the deterioration of their condition or transfer to other hospitals, which might affect the patients' selection for our analyses.

## Conclusion

Our study suggested that lipid levels at the presentation of ICH were associated with 3-month prognosis in women, especially when the TG <135.56, TC <193.50, LDLC <135.06, non-HDLC <156.54, and HDLC > 36.96 mg/dl. Low levels of TG, TC, LDLC, non-HDLC, and high levels of HDLC are independently associated with the poor prognosis of spontaneous ICH in women.

## Data Availability Statement

The original contributions presented in the study are included in the article/supplementary material, further inquiries can be directed to the corresponding author/s.

## Ethics Statement

The studies involving human participants were reviewed and approved by the study protocol was approved by the Institutional Review Board of the Beijing Tiantan Hospital affiliated to Capital Medical University and the Beijing Tiantan Hospital Ethics Committee approved this study. The patients/participants provided their written informed consent to participate in this study.

## Author Contributions

HF, XW, and XZ designed the study and drafted the manuscript. HF and WW collected the clinical data and managed the database. All authors approved for the manuscript submitted.

## Conflict of Interest

The authors declare that the research was conducted in the absence of any commercial or financial relationships that could be construed as a potential conflict of interest.
